# Gastric Tube Insertion Performance of Second-Generation Supraglottic Airway Devices: A Prospective Manikin Study With Literature Review

**DOI:** 10.7759/cureus.67863

**Published:** 2024-08-26

**Authors:** Yoshiya Adachi, Mineto Kamata, Akira Kitamura

**Affiliations:** 1 Department of Anesthesiology, Saitama Medical University International Medical Center, Saitama, JPN

**Keywords:** lma proseal, gastric tube, second-generation supraglottic airway devices, i-gel, lma-supreme, ambu auragain

## Abstract

Background: Second-generation supraglottic airway devices (SGAs) are pivotal in airway management, with the ability to accommodate gastric tube (GT) insertion. However, research on GT insertion with SGAs under controlled conditions is limited. This study aimed to evaluate the GT insertion performance of SGAs using a manikin.

Methods: This prospective study included 32 anesthesiologists in our department, each with more than two years of clinical experience. These anesthesiologists randomly inserted four second-generation SGAs, including i-gel (Intersurgical Ltd., Berkshire, UK), Ambu AuraGain (Ambu, Copenhagen, Denmark), LMA ProSeal (Teleflex Medical, Wayne, PA), and LMA Supreme (Teleflex Medical), all of size 4, into a manikin, followed by GT insertion using Salem Sump™ tubes (Cardinal Health, Dublin, OH) (12 Fr for i-gel and 14 Fr for others) until the GT was 55 cm deep at the port entrance. The primary outcome was the difference in GT insertion time, with participants' evaluations.

The usual use of second-generation SGAs, including GT insertion, was also surveyed. The differences in GT insertion time among the four SGAs were analyzed using the Friedman test, followed by the Bonferroni method for post-hoc analysis. P < 0.05 was considered significant.

Results: The median GT insertion times were 17.2 seconds for i-gel, 9.9 seconds for AuraGain, 18.8 seconds for ProSeal, and 8.9 seconds for Supreme. These times showed significant differences (p < 0.001). Post-hoc analysis revealed that both Supreme and AuraGain had significantly shorter insertion times than i-gel and ProSeal, respectively (p < 0.001). Of the participants, 59% (19/32) evaluated Supreme as the easiest SGA for GT insertion, which is consistent with the observed insertion times. i-gel was the most frequently used SGA, chosen by 72% (23/32) of participants. Additionally, 72% (23/32) of anesthesiologists inserted GTs less than half as often following second-generation SGA placement.

Conclusions: Significant differences in GT insertion performance were found among the four second-generation SGAs. According to a survey of participants, second-generation SGAs were often used without GT insertion. Although the differences between products may not be clinically significant, selecting an SGA with easy GT insertion may improve the efficiency and reliability of gastric content drainage and enhance the safety of airway management when using SGAs.

## Introduction

Supraglottic airway devices (SGAs) have become indispensable in airway management, particularly in the management of difficult airways [1]. While airway management performance is the most important factor for SGAs, the advent of second-generation SGAs, which facilitate gastric tube (GT) placement through the addition of a GT port and enhance oropharyngeal leak pressure, has led to their widespread adoption as alternatives to tracheal intubation, even in laparoscopic surgery settings [2]. For optimal safety, it is recommended to utilize second-generation SGAs with superior protection against gastric insufflation and aspiration [1].

Although many studies have focused on the ventilation performance of second-generation SGAs, few have prioritized the evaluation of GT insertion performance despite it being an important feature of these devices. Furthermore, most studies examining GT insertion performance have not precisely evaluated the performance under controlled conditions because the type and size of GTs used were not standardized or described. This study aimed to assess the GT insertion performance of second-generation SGAs, focusing on insertion time and participant evaluation using a manikin. Additionally, previous studies comparing GT insertion performance among second-generation SGAs were comprehensively reviewed, with a particular focus on insertion time via the SGAs.

## Materials and methods

Study institution and ethics

This prospective study was conducted in the Department of Anesthesiology, Saitama Medical University International Medical Center, Japan. This study was approved by the Ethics Committee of the center (approval number: 2021-2023; approval date: January 4, 2022), and written consent for the research was obtained from all participants.

SGAs, GTs, and airway manikin

**Figure 1:**
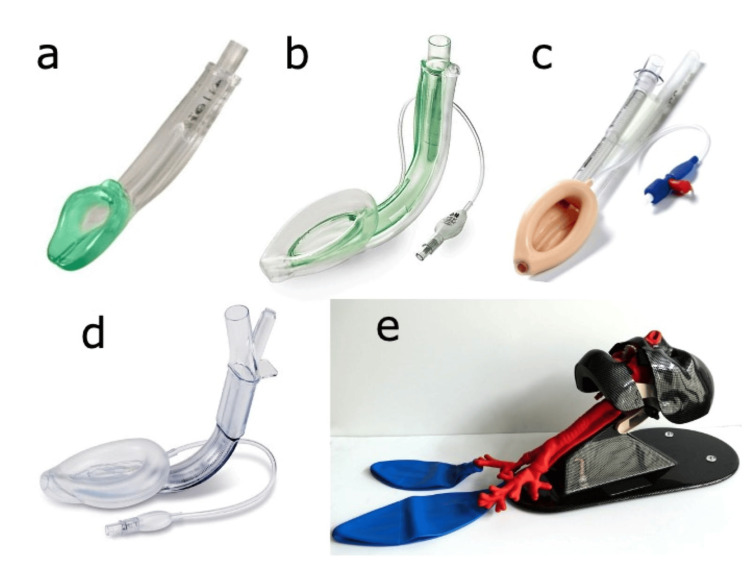
Supraglottic airway devices and airway manikin. (a) i-gel®; (b) Ambu® AuraGain™; (c) LMA® ProSeal™; (d) LMA® Supreme™; (e) TruCorp AirSim®.

In this study, four second-generation SGAs were compared, including i-gel® (Intersurgical Ltd., Berkshire, UK), Ambu® AuraGain™ (Ambu, Copenhagen, Denmark), LMA® ProSeal™ (Teleflex Medical, Wayne, PA), and Supreme™ (Teleflex Medical), all in size #4, which are commonly used in clinical practice in Japan. The size of the GTs (Salem Sump™, Cardinal Health, Dublin, OH) was selected according to the manufacturer’s instructions. The recommended sizes for i-gel and Supreme were 12 Fr and 14 Fr, respectively. A 16-Fr GT was recommended for AuraGain and ProSeal. However, based on preliminary findings indicating difficulty and prolonged insertion time with the recommended sizes of AuraGain and ProSeal, a 14-Fr GT was chosen. TruCorp AirSim (AirSim®, TruCorp Ltd., Craigavon, UK) manikin was utilized for this study (Figure 1).

Participants

Thirty-two anesthesiologists in our department, each with more than two years of clinical experience, participated in our study.

Study protocol

Each participant inserted all four SGAs and GTs into the airway manikin, with the order of the SGAs randomized using a computer-generated sequence of random numbers. The study was conducted in the operating room where the ambient temperature was maintained at 25°C.

SGA placement and GT insertion

Participants inserted the SGAs following the manufacturer’s instructions. Subsequently, ventilation was established by connecting a self-inflating bag (SPUR®, Ambu), and its completion was verified through a visual assessment of the manikin’s lung extension. Following SGA placement, participants were instructed to insert a correctly sized and well-lubricated GT through each GT port. Completion of GT insertion was determined by reaching a depth of 55 cm at the port entrance, verified through direct visual inspection of the manikin’s esophageal outlet. GT insertion time was measured from when the participant was prepared to insert the GT until insertion was completed.

Water-soluble lubricating jelly (Cathe Jelly^TM^, BD Biosciences, Franklin Lakes, NJ) was used as a lubricant for the insertion of SGAs and GTs. All SGAs used in this study were new, while GTs were reused multiple times; however, the lubricant jelly was washed off, and the tubes were dried before each trial. During SGA and GT insertion, the head position of the airway manikin was fixed in extension.

Questionnaire

After GT insertion, participants were asked to assess and rank the ease of GT insertion for each SGA, providing a sequential order based on their perceived ease of use. Additionally, a survey was conducted to gather information on the routine utilization of second-generation SGAs, selection criteria, and the frequency of GT insertion when using these devices.

Outcomes

The primary outcome was the GT insertion performance among the four second-generation SGAs based on GT insertion time and questionnaire. Secondary outcomes included the correlation between GT insertion time and anesthesiologist experience with each SGA, as well as the utilization of GTs when using second-generation SGAs.

Literature review

The PubMed database was searched using the keywords “supraglottic airway device,” “i-gel,” “Ambu AuraGain,” “LMA ProSeal,” and “LMA Supreme” to identify relevant studies comparing GT insertion time with second-generation SGAs. Studies published between May 2001 and December 2023 were included. Additionally, the references of relevant articles were further searched to ensure a comprehensive literature review.

Statistical analysis

Data are expressed as median (interquartile range, IQR), range, or frequency. Data distribution was assessed using the Shapiro-Wilk test. Differences between products were analyzed using the Friedman test. Post-hoc analysis was performed using the Bonferroni method. Correlations between GT insertion time with each SGA and the anesthesiologist’s experience were evaluated using Spearman’s rank correlation. Statistical analyses were conducted using R software version 4.2.0 (R Foundation for Statistical Computing, Vienna, Austria). Statistical significance was set at p < 0.05.

## Results

All 32 participants completed the placement of the SGAs, GT insertion, and questionnaire. The participating anesthesiologists had a median of 11 years of experience (IQR: 6, 23; range: 3-36). Median GT insertion times were 17.2 seconds (12.3, 26.7) with i-gel, 9.9 seconds (9.3, 11.9) with AuraGain, 18.8 seconds (12.8, 33.4) with ProSeal, and 8.9 seconds (7.1, 11.7) with Supreme. The Friedman test indicated significant differences among the four SGAs (p < 0.001). Post-hoc analysis revealed that Supreme and AuraGain exhibited significantly shorter GT insertion times than i-gel and ProSeal (p < 0.001), respectively. Additionally, no correlation was found between the time with each SGA and the anesthesiologist's experience (Table 1).

**Table 1:** Main results of the study. Data are shown as median (interquartile range) or Spearman’s rank correlation coefficient and p-value. ^*^ The Friedman test was utilized to assess the statistical significance of differences among the four SGAs. ^†^ p < 0.001 versus i-gel and ProSeal assessed by Bonferroni test. ^‡^ Spearman’s rank correlation coefficient was utilized to assess the correlation. GT, gastric tube; N/A, not applicable; SGA, supraglottic airway device.

SGA (# size)	GT size	GT insertion time	The correlation coefficient between GT insertion time and the anesthesiologist’s experience^‡^
i-gel (#4)	12 Fr	17.2 (12.3, 26.7) seconds	0.0515, p = 0.78
AuraGain (#4)	14 Fr	9.9 (9.3, 11.9) seconds^†^	-0.131, p = 0.476
ProSeal (#4)	14 Fr	18.8 (12.8, 33.4) seconds	-0.279, p = 0.122
Supreme (#4)	14 Fr	8.9 (7.1, 11.7) seconds​​​​​​​^†^	0.0438, p = 0.812
p-value		<0.001^*^	N/A

The questionnaire results are shown in Table 2. According to the questionnaire responses, 59% (19/32) of participants rated Supreme as the easiest SGA for GT insertion among the four SGAs, followed by AuraGain (25%, 8/32), which is consistent with the insertion time. Conversely, 47% (15/32) of participants evaluated ProSeal as the most challenging SGA for GT insertion, followed by i-gel (44%, 14/32). The most used SGA was the i-gel (72%, 23/32), followed by ProSeal (15%, 5/32). While the reasons for selecting the i-gel varied, none of the anesthesiologists cited the possibility of GT insertion as a contributing factor.

**Table 2:** Results of the questionnaire (n = 32). Data are presented as number (%), median (interquartile range), and range. GT, gastric tube; N/A, not applicable; SGA, supraglottic airway device.

Survey questions	SGA	n	%
Second-generation SGA with the easiest GT insertion	Supreme	19/32	59%
	AuraGain	8/32	25%
	i-gel	2/32	6%
	ProSeal	2/32	6%
	N/A	1/32	3%
Second-generation SGA with the most challenging GT insertion	Supreme	2/32	6%
	AuraGain	0/32	0%
	i-gel	14/32	44%
	ProSeal	15/32	47%
	N/A	1/32	3%
SGA in regular use	i-gel	23/32	72%
	ProSeal	5/32	15%
	Other	4/32	13%
Frequency of GT insertion when using second-generation SGA	100–75%	9/32	28%
	75–50%	0/32	0%
	50–25%	7/32	22%
	0–25%	16/32	50%
Frequency of GT insertion when i-gel is used (respondents who answered i-gel as their primary choice) (n = 23)	100–75%	5/23	22%
	75–50%	0/23	0%
	50–25%	12/23	52%
	0–25%	6/23	26%

In response to a survey question regarding GT insertion when using second-generation SGAs in daily clinical practice, 72% (23/32) of the anesthesiologists indicated that the frequency of GT insertion following SGA placement was less than half of their routine practice. Of the anesthesiologists, 78% (18/23) who used the i-gel as their first choice also responded that they did not insert a GT when using the i-gel.

Our literature review identified nine studies that primarily examined GT insertion performance, specifically focusing on GT insertion time [3-11]. In all studies, SGA ventilation performance served as the primary outcome measure. Only one study specified the product and size of the GT used.

## Discussion

This is the first study to evaluate GT insertion performance among four second-generation SGAs, focusing on GT insertion performance while specifying the GT type used. Our study found that AuraGain and Supreme exhibited superior GT insertion performance, aligning with findings reported in prior research.

Of the nine studies [3-11] identified in the literature review (Table 3), only one study explicitly specified the size and type of GT used to evaluate GT insertion performance as a secondary outcome [3]. Singh et al. compared GT insertion times among three second-generation SGAs, Supreme, i-gel, and ProSeal, describing the size of the GT and the specific product name; 14-Fr Ryle’s tubes were utilized for Supreme and ProSeal, and 12-Fr for i-gel. The reported GT insertion times were as follows: 9.0 ± 2.78 seconds, 12.21 ± 3.82 seconds, and 8.89 ± 2.58 seconds for Supreme, i-gel, and ProSeal, respectively, indicating significantly shorter times for Supreme and ProSeal than for i-gel [3]. Similarly, Lopez et al. compared the GT insertion performance of three SGAs, including AuraGain, Supreme, and i-gel, using cadavers [4]. AuraGain and Supreme utilized a 16 Fr GT, while i-gel employed a 12-Fr or 14-Fr GT; however, specific product names were not provided. The findings indicated that the insertion time was approximately half for AuraGain and Supreme compared to i-gel (AuraGain: 5 ± 3 seconds, Supreme: 6 ± 2 seconds, i-gel 12 Fr: 12 ± 4 seconds, i-gel 14 Fr: 12 ± 10 seconds). Although the study lacked statistical analyses, the reported results align with those of our study.

**Table 3:** Studies comparing gastric tube insertion times in second-generation SGAs. Data are shown as mean ± standard deviation, median (interquartile range), and mean (range). A, AuraGain; ETT, endotracheal tube; GT, gastric tube; i, i-gel; NMB, neuromuscular block; N/A, not applicable; P, ProSeal; S, Supreme; SGA, supraglottic airway device; ASA-PS, American Society of Anesthesiologists physical status.

Author and reference	Comparison	SGA #size (GT: size, product)	GT insertion time	Other evaluation
Singh et al. [3]	ASA-PS I, II	i-gel #3–5 (12 Fr, Ryle’s tube)	i: 12.2 ± 3.8 seconds	All GT insertions were “easy”
	18–60 years	Supreme #3–5 (14 Fr, Ryle’s tube)	S: 9.0 ± 2.8 seconds	
	n = 84	ProSeal #3–5 (14 Fr, Ryle’s tube)	P: 8.9 ± 2.6 seconds	
	NMB (+)		Significant	
Lopez et al. [4]	Fresh cadaver	i-gel #3/4, 5 (12/14 Fr, N/A)	i: 12 Fr = 12 ± 4 seconds, 14 Fr = 12 ± 10 seconds	Ease of GT insertion “easy”
	n = 7	AuraGain #3–5 (16 Fr, N/A)	A: 5 ± 3 seconds	A, P: 100% vs. i: 43%
		Supreme #3–5 (16 Fr, N/A)	S: 6 ± 2 seconds	No statistical comparison
Teoh et al. [5]	ASA-PS I, II	i-gel #3 or 4 (12 Fr, N/A)	i: 15.1 ± 7.3 seconds	Ease of GT insertion “easy”
	21–80 years	Supreme #3 or 4 (14 Fr, N/A)	S: 9.0 ± 2.5 seconds	i: 78% vs. S: 100% (significant)
	Gynecological laparoscopic surgery		Significant	Amount of gastric contents aspirated at GT placement (not significant)
		n = 100			
	NMB (+)			
	Fernández Díez et al. [6] (Only abstract available)	Major outpatient	i-gel N/A (N/A)		i: 22.1 seconds	Success rate of GT insertion
Surgery under general anesthesia		Supreme N/A (N/A)	S: 9.5 seconds	i: 85.7% vs. S: 97.6%
n = 85			Mean, significant	
NMB (-)				
Park et al. [7]	Laparoscopic	i-gel #3 or 4 (12 Fr, N/A)	i: 20.4 ± 3.9 seconds	All GTs were successfully inserted on the first attempt
	Cholecystectomy	Supreme #3–5 (14 Fr, N/A)	S: 16.7 ± 1.6 seconds	
	n = 93		Significant	
	ASA I, II			
	NMB (+)			
Shariffuddin et al. [8]	ASA-PS I-III	AuraGain #3 or 4 (14 Fr, N/A)	A: 18.9 ± 5.8 seconds	Success rate of GT insertion
	n = 93	Supreme #3 or 4 (14 Fr, N/A)	S: 18.7 ± 4.9 seconds	A: 100% vs. S: 90.9% (significant)
	NMB (-)		Not significant	Ease of GT insertion “easy”
				A: 75.5% vs. S: 61.4% (not significant)
Hur et al. [9]	Simulated cervical	i-gel #3–5 (10–14 Fr, N/A)	i: 10 (9, 15) seconds	Number of GT insertion attempts
	Immobilization	AuraGain #3–5 (12 or 14 Fr, N/A)	A: 8 (7, 10) seconds	Not significant
	Patients		Significant	Ease of GT insertion “easy”
	n = 104			Not significant
	NMB (+)			
Hosten et al. [10]	Elective surgery	Supreme #3–5 (16 Fr, N/A)	S: 10.3 ± 2.2 seconds	Success rate of GT insertion
	ASA-PS I, II	ProSeal #3–5 (16 Fr, N/A)	P: 15.2 ± 5.5 seconds	S: 100% vs. P: 80%
	≥18 years		Significant	Significant
	n = 60			
	NMB (-)			
Borkowski et al. [11] (Only abstract available)	Gynecological	ETT	ETT: 57 (22–219) seconds	Ease of GT insertion (not significant)
	Laparoscopic surgery	ProSeal N/A (N/A)	P: 38 (15–75) seconds	
	n = 65		Significant	
	NMB (+)			

Here, Supreme demonstrated the most favorable outcomes in terms of GT insertion time and questionnaire responses. Supreme is equipped with a large-bore drain tube integrated into the posterior aspect of the airway, serving as a dedicated GT port. The drain tube is centrally oriented and opens at the distal end of the cuff, facilitating smooth GT insertion. In our study, Supreme demonstrated GT insertion without encountering resistance. In contrast, the i-gel features a narrow GT port, with the drain tube running slightly horizontally within the cuff before opening at the device’s tip. Throughout our study, a significant number of participants reported encountering resistance during GT insertion, particularly around the point where the i-gel was bent, approximately 15 cm from the port entrance. Given these findings, it is reasonable that numerous studies have consistently demonstrated the superior capability of Supreme in facilitating the swifter insertion of larger-sized GT than i-gel [3,5-7].

AuraGain, similar to Supreme, features an anatomically curved airway tube and an integrated large-diameter GT channel. Some reports indicate that Supreme and AuraGain performed comparably regarding GT insertion [8,12]. AuraGain was reported to facilitate faster GT insertion than i-gel [4,9].

The path of ProSeal’s drainage tube resembles that of Supreme, running parallel to the airway tube and centrally extending beyond the airway opening to eventually open at the device’s tip center. Hosten et al. [10] demonstrated the superior efficacy of Supreme over ProSeal, using a 16-Fr GT, regarding success rate and insertion time. Their results are consistent with our findings, showing a shorter insertion time (Supreme: 10.3 ± 2.2 seconds, ProSeal: 15.2 ± 5.5 seconds, p < 0.001) [10]. In a meta-analysis of 12 randomized clinical trials, Tan et al. compared the first-time success rate of GT insertion between ProSeal and i-gel and found no significant difference (relative risk: 1.04, 95% CI: 0.99, 1.10, p = 0.11). They concluded that the performance of GT insertion with ProSeal and i-gel was comparable [13].

Two factors could contribute to the difference in GT insertion performance. First, the curvature of the SGA plays a significant role. Supreme and AuraGain, being angle-type SGAs, demonstrated superior performance in this study. The built-in drain tubes of Supreme and AuraGain are designed to conform to the curvature of the pharynx, minimizing shape changes of the GT port after placement. In contrast, i-gel and ProSeal are straight-type SGAs, resulting in curvature during SGA placement. This could deform the lumen of the GT port. Given these factors, angle-type SGAs offer advantages for GT insertion, as observed in our study. The second factor to consider is the material composition of the gastric port. AuraGain and Supreme use polyvinyl chloride for the gastric ports, known for their low coefficients of friction. Additionally, the AuraGain port is internally coated with a low-friction surface, further reducing resistance during GT insertion. In contrast, the i-gel gastric port, made of styrene polymer, is created by hollowing out the body of the port. ProSeal features an independent gastric drainage tube made of silicone, which exhibits high friction resistance. Given these factors, the material composition within the GT port significantly influences the ease and performance of GT insertion.

The questionnaire results indicated that GT insertion did not significantly affect the choice for second-generation SGAs. Among the SGAs evaluated, i-gel, the only SGA not requiring cuff adjustment, consistently remained the preferred first choice. This highlights the importance of ease and speed in securing the airway when selecting an SGA. It is assumed that SGAs are primarily used when the aspiration risk is low and the need for GT insertion is minimal.

When using an SGA, GT insertion is advisable. While aspiration of gastric contents is uncommon in patients who observe fasting before elective surgery, it becomes crucial in obese patients and those undergoing laparoscopic procedures to prevent aspiration and secure the operative field [14]. This is because positive pressure ventilation during high inspiratory pressure application with an SGA can lead to air entering the stomach. Using a GT with active or passive drainage is beneficial for ongoing gastric decompression, thereby mitigating the risk of aspiration. García-Aguado et al. highlighted another benefit of GT insertion during airway management with SGA. They observed that inserting a GT before SGA placement enhances the success rate of SGA insertion [15]. In anecdotal reports, López et al. noted that the insertion of a GT facilitates SGA reinsertion if it is accidentally removed while the patient is in a prone position [16].

This study has limitations. First, it was conducted at a single institution, which may have introduced bias based on the anesthesiologist’s experience and usual practice. Although all participants had sufficient experience with SGAs, the results may not fully represent the outcomes in different clinical settings or with less experienced practitioners. Second, the size of the GTs used may have significantly affected the insertion time. Lastly, the manikin model used in this study may be more difficult to insert GTs than other models [17].

## Conclusions

Significant differences in GT insertion performance were observed among the four second-generation SGAs, with AuraGain and Supreme demonstrating better performance. These differences may be attributed to variations in SGA design, including factors such as the material composition of the GT port and device curvature. According to the survey of participants, second-generation SGAs were often used without GT insertion. Although the differences between products may not be clinically significant, choosing SGAs with easy GT insertion could improve the efficiency and reliability of gastric contents drainage and enhance the safety of airway management when using SGAs. Future studies should aim to refine techniques for enhancing the ease and success rate of GT insertion through SGAs to improve patient outcomes in airway management.
